# Multivariate Tail Probabilities: Predicting Regional Pertussis Cases in Washington State

**DOI:** 10.3390/e23060675

**Published:** 2021-05-27

**Authors:** Xuze Zhang, Saumyadipta Pyne, Benjamin Kedem

**Affiliations:** 1 Department of Mathematics and Institute for Systems Research, University of Maryland, College Park, MD 20742, USA; xzhang51@umd.edu; 2 Public Health Dynamics Laboratory, Department of Biostatistics, Graduate School of Public Health, University of Pittsburgh, Pittsburgh, PA 15261, USA; 3 Health Analytics Network, Pittsburgh, PA 15237, USA

**Keywords:** disease outbreak, density ratio model, variable tilt, model selection, goodness-of-fit, data fusion

## Abstract

In disease modeling, a key statistical problem is the estimation of lower and upper tail probabilities of health events from given data sets of small size and limited range. Assuming such constraints, we describe a computational framework for the systematic fusion of observations from multiple sources to compute tail probabilities that could not be obtained otherwise due to a lack of lower or upper tail data. The estimation of multivariate lower and upper tail probabilities from a given small reference data set that lacks complete information about such tail data is addressed in terms of pertussis case count data. Fusion of data from multiple sources in conjunction with the density ratio model is used to give probability estimates that are non-obtainable from the empirical distribution. Based on a density ratio model with variable tilts, we first present a univariate fit and, subsequently, improve it with a multivariate extension. In the multivariate analysis, we selected the best model in terms of the Akaike Information Criterion (AIC). Regional prediction, in Washington state, of the number of pertussis cases is approached by providing joint probabilities using fused data from several relatively small samples following the selected density ratio model. The model is validated by a graphical goodness-of-fit plot comparing the estimated reference distribution obtained from the fused data with that of the empirical distribution obtained from the reference sample only.

## 1. Introduction

A challenging statistical problem is the estimation of lower and upper tail probabilities from a given small data set. Challenging as it is, the problem becomes even more arduous when the data set lacks information about lower or upper tail data to the extent that the use of the empirical distribution becomes problematic. This calls for additional data in some form. In this study, the fusion of data from multiple sources allows us to compute tail probabilities, which could not otherwise be obtained due to the lack of lower or upper tail data.

In particular, if the data from a certain source exceed a sufficiently high threshold, then information about lower values below the threshold can be obtained by fusion with other sources that do have data below the threshold. The same holds for sources with data below a given threshold. This necessitates fusion with data sources containing data above the threshold. Our approach is particularly useful when the sample sizes are relatively small and yet probabilities of unusual or extreme values are of interest [1]. Here, we present a multivariate extension of our methodology and demonstrate its application using small pertussis case count data sets.

Pertussis, or whooping cough, is an acute infectious disease of the respiratory tract caused by the gram-negative bacterium *Bordetella pertussis*. It is highly contagious and transmitted from infected to susceptible individuals by airborne droplets due to coughing and sneezing. Pertussis affects people of all ages but can be serious in infants less than 1 year of age and causes 195,000 infant deaths annually, mostly in developing countries. The global estimates in 2014 were 24.1 million cases and 160,700 deaths from the disease among children below five years of age [2]. Pertussis is endemic in all countries and tends to occur every two to five years in North America and Europe [3].

After widespread vaccination began in the U.S. in the 1940s, the number of new infections reduced to 10,000–40,000 cases of pertussis reported each year, resulting in a 100-fold reduction in the incidence of the disease, thereby making it a likely candidate for elimination. However, since the mid 1970s, pertussis incidence has steadily increased [4]. In 2012, 48,277 pertussis cases were reported in the U.S. (an incidence rate of 15.1 per 100,000), the largest number since 1955 [5]. In Washington state alone, more than 4600 pertussis cases were reported in 2012, mostly among infants aged less than 1 year and children aged 10 years [6]. The incidence of the disease among adolescents of age 13–14 years and adults has also increased, including those previously vaccinated, suggesting early waning of vaccine-acquired immunity.

While vaccination remains the most effective means of preventing illness, pertussis has re-emerged in countries that have sustained high vaccine coverage. In the U.S., pertussis has been a reportable disease since 1922, and case-based surveillance data are available through the National Notifiable Diseases Surveillance System (NNDSS) of the Centers for Disease Control and Prevention (CDC) and, additionally, the Enhanced Pertussis Surveillance (EPS) in seven states [7]. The reasons for this re-emergence are attributable to several factors including changes in diagnostic testing and reporting, increased awareness, mismatch of vaccine antigens and circulating strains, reduced duration of immunity from acellular pertussis (aP) vaccines that replaced whole-cell vaccines in the U.S. during the 1990s, and changes in the *B. pertussis* organism at the molecular level [7].

During the 2012 pertussis outbreak in Washington state, it was observed that the incidence was highest in infants of age <1 year and children of age 10, 13 and 14 years [6]. The statewide incidence rate was higher among Hispanics than non-Hispanics [6]. Household size [7] and vaccination coverage [8] have been considered among the risk factors of the disease. We have noted such risk factors in Table A1.

Apart from the analysis of factors that affect the resurgence of pertussis, forecasting upper and lower joint tail probabilities of high incidence in a given period of time is another key topic of interest to epidemiologists. While a variety of methods for modeling pertussis incidence have been proposed in recent years [9,10], here we present a method for the forecasting of both univariate as well as multivariate joint tail probabilities using the fusion of pertussis count data obtained from neighboring counties in Washington state. Our approach is based on the so-called *density ratio model with variable tilts* presented here with a multivariate extension, which is the novel contribution of this study.

## 2. Density Ratio Model

Given m+1 independent *p*-dimensional multivariate random samples $X_{k}=\left\{X_{k1},\ldots ,X_{kn_{k}}\right\}$, k=0,…,m, where $n_{k}$’s are the corresponding sample sizes. Suppose that $X_{k}$ has a density $g_{k}$ for k=0,…,m, where the $g_{k}$ satisfy the density ratio structure

(1) $$\frac{g_{k}\left(x\right)}{g_{0}\left(x\right)}=\exp\left(\alpha_{k}+\beta_{k}^{T}h_{k}\left(x\right)\right)\;k=1,\ldots ,m,$$

where $h_{k}$ is referred to as a tilt functions or simply tilt. The sample $X_{0}$ is referred to as the reference sample and the rest of the samples are referred to as tilted samples.

Let $\alpha ={\left(\alpha_{1},\ldots ,\alpha_{m}\right)}^{T}$, $\beta ={\left(\beta_{1}^{T},\ldots ,\beta_{m}^{T}\right)}^{T}$ and $\theta ={\left(\alpha^{T},\beta^{T}\right)}^{T}$. Let $w_{0}\left(\cdot ;\theta \right)=1$ and $w_{k}\left(\cdot ;\theta \right)=\exp\left(\alpha_{k}+\beta_{k}^{T}h_{k}\left(\cdot \right)\right)$. Denote the combined sample by $t=\left\{t_{1},\ldots ,t_{n}\right\}=\left\{X_{01},\ldots ,X_{0n_{0}},\ldots ,X_{m1},\ldots ,X_{mn_{m}}\right\}$ with the corresponding samples size $n=n_{0}+\ldots +n_{m}$. Let $G_{0}$ be the reference cumulative distribution function that corresponds to the density $g_{0}$. The empirical likelihood function can be written as

(2) $$L\left(\theta ;G_{0}\right)=\prod_{i=1}^{n}p_{i}\prod_{k=1}^{m}\prod_{j=1}^{n_{k}}w_{k}\left(X_{kj};\theta \right)$$

with constraints

$$\sum_{i=1}^{n}p_{i}=1\;\sum_{i=1}^{n}p_{i}\left[w_{k}\left(t_{i};\theta \right)-1\right]=0\;k=1,\ldots ,m,$$

where $p_{i}$ is the jump of $G_{0}$ at $t_{i}$. By profiling, the $p_{i}$’s that maximize the empirical likelihood are given by

$$p_{i}=\frac{1}{\sum_{k=0}^{m}n_{k}w_{k}\left(t_{i};\theta \right)}.$$

Therefore, the likelihood becomes a function of θ only and we can find the estimator $\tilde{\theta }$ that maximizes the likelihood. Subsequently, the estimator of $p_{i}$ is obtained as

$${\tilde{p}}_{i}=\frac{1}{\sum_{k=0}^{m}n_{k}w_{k}\left(t_{i};\tilde{\theta }\right)}.$$

It can be shown that $\tilde{\theta }$ has the asymptotic normal distribution

(3) $$\sqrt{n}\left(\tilde{\theta }-\theta_{0}\right)\overset{d}{\to }N\left(0,\Sigma \left(\theta_{0}\right)\right)$$

as n→∞. Details can be found in [11,12,13,14,15].

The estimated $G_{0}$ is obtained from the accumulation of the ${\tilde{p}}_{i}$’s,

(4) $${\tilde{G}}_{0}\left(\left(x_{1},\ldots ,x_{p}\right)\right)=\sum_{i=1}^{n}{\tilde{p}}_{i}I\left[t_{i1}\leq x_{1},\ldots ,t_{ip}\leq x_{p}\right].$$

In the above expression for ${\tilde{G}}_{0}$, replacing ${\tilde{p}}_{i}$ by 1/n we get the reference empirical distribution ${\hat{G}}_{0}$.

The selection of the tilts $h_{k}$’s can be based on [16,17,18].

**Figure 1:**
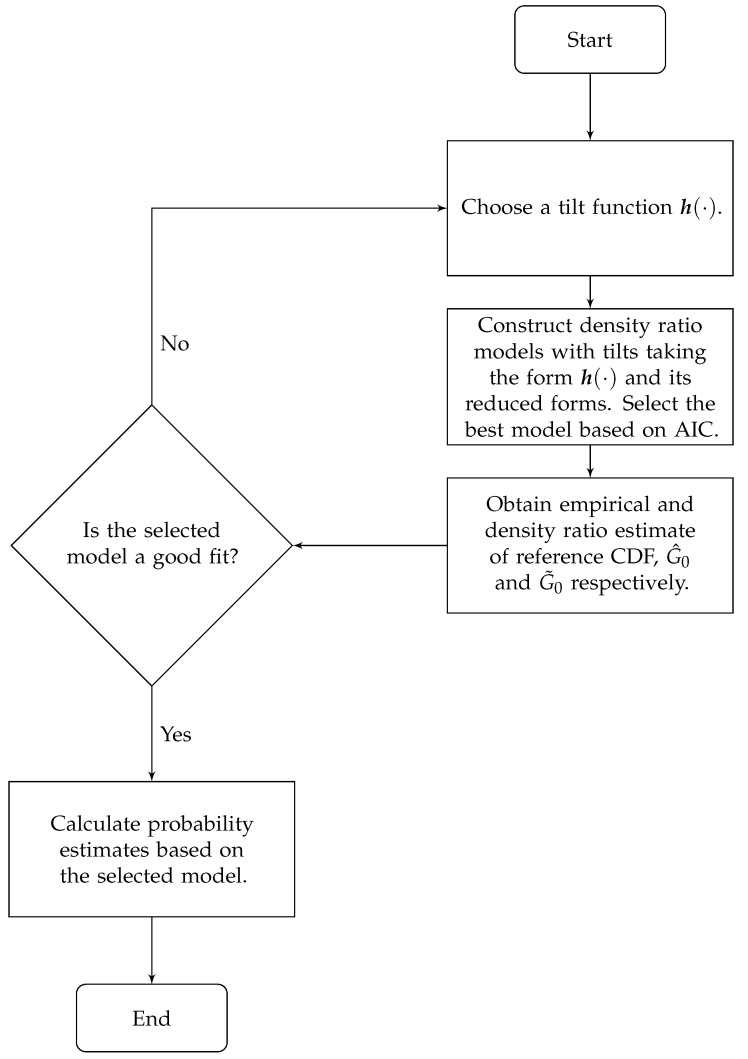
Flowchart of the data fusion analysis.

A flowchart in Figure 1 is provided to illustrate the steps in the data fusion analysis.

## 3. Application: County-level Pertussis Cases in Washington State

We collected Washington state county-level annual data of the number of pertussis cases from 1997–2018 (Washington Department of Health Website https://www.doh.wa.gov/ (accessed on 1 March 2021)). For each county, we have a sample of size 22. Without any distribution assumption, when county tail data are available we can estimate tail probabilities from the empirical distribution. However, such an estimation is not feasible if tail data are absent. For example, from Table 1 we see that no observation exceeds 30 in Jefferson county so that estimating the chance of exceeding the threshold of 30 from the empirical distribution is not viable.

Nevertheless, the estimation of this probability is possible via the density ratio model if we fuse the sample from Jefferson county with samples from the counties of Cowlitz and Snohomish for which sufficient amounts of data above 30 are available.

### 3.1. Univariate Analysis

The sample from 0-Jefferson is taken as the reference while the samples from 1-Cowlitz and 2-Snohomish are tilted with tilts $h_{1}\left(x\right)=h_{2}\left(x\right)=x$ as suggested in [14]. Using the fused data from the three counties, and appealing to the density ratio model, tail probabilities for Jefferson County are given in Table 2 for thresholds 30, 40 and 50. As discussed above, these tail probabilities cannot be estimated by the empirical distribution for lack of tail data.

**Figure 2:**
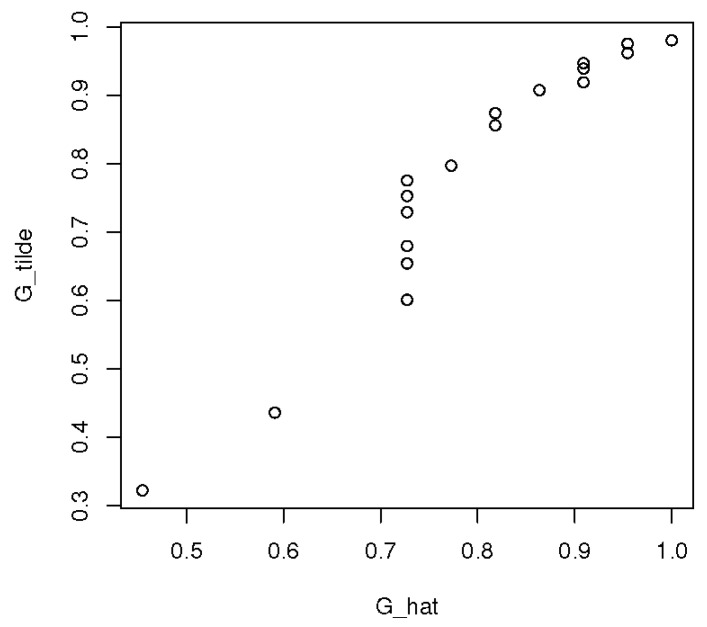
PP-plot for ${\hat{G}}_{0}$ vs. ${\tilde{G}}_{0}$ in the univariate case.

To validate the model, we used the graphical goodness-of-fit discussed in [15]. The idea is to see whether the points (${\hat{G}}_{0}$,${\tilde{G}}_{0}$) lie on or close to a 45°-line. From the goodness-of-fit graph in Figure 2, we see that some points lie not far from a 45°-line while others do not, pointing to a possible lack of fit. Moreover, little improvement has been observed by using different tilt functions. To resolve this issue as to the suitability of the density ratio model, we turn to the multivariate version of the model, where a somewhat *richer class of possible tilts* is used. This leads to, as we shall see in the next section, remarkable improvement in the fit.

### 3.2. Multivariate Analysis

We took 3-dimensional (that is p=3) samples from three different regions: 0-(Grays Harbor, Jefferson, Clallam), 1-(Clark, Cowlitz, Lewis), 2-(King, Snohomish, Skagit). The order for each region is from the most to the least populated. Therefore, we obtained three 3-dimensional multivariate random samples with sample sizes all equal to 22 where the sample from (Grays Harbor, Jefferson, Clallam) was considered as the reference sample. The summary statistics of the nine counties are shown in Table 3.

**Figure 3:**
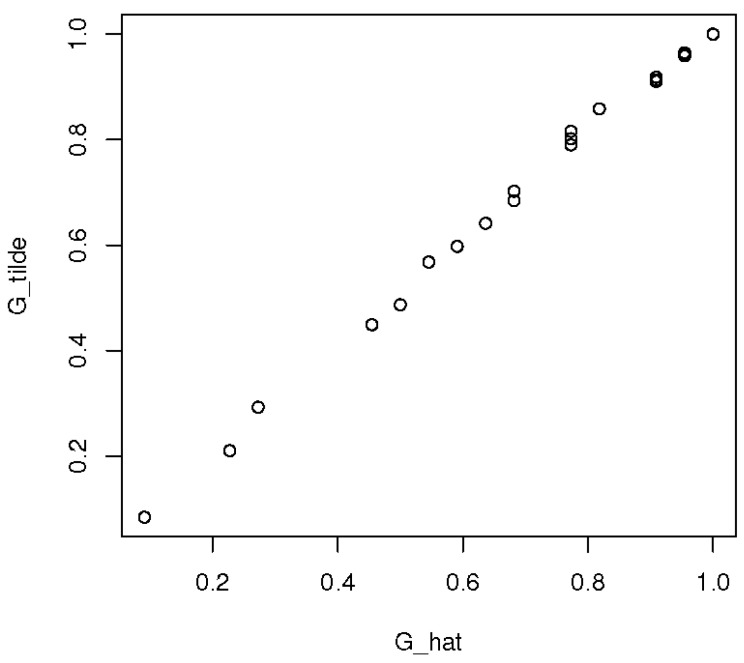
PP-plot for ${\hat{G}}_{0}$ vs. ${\tilde{G}}_{0}$ in the univariate case.

We initiated tilt selection with $h_{1}\left(x\right)=h_{2}\left(x\right)={\left(x_{1},x_{2},x_{3}\right)}^{T}$ suggested in [14,15]. The tilts selected were $h_{1}\left(x\right)={\left(x_{1},x_{2},x_{3}\right)}^{T}$ and $h_{2}\left(x\right)={\left(x_{1},x_{3}\right)}^{T}$ giving the smallest AIC = 483.22 as shown in Table 4. The 45°-line formed by the pairs (${\hat{G}}_{0}$,${\tilde{G}}_{0}$) in Figure 3 indicating a good fit (${\tilde{G}}_{0}$ is closed to the empirical distribution ${\hat{G}}_{0}$).

We computed in Table 5 the estimates of several selected joint threshold probabilities, which can be regarded as predictions for a future year. It is worth noticing that the probabilities selected cannot be estimated by the empirical distribution ${\hat{G}}_{0}$ due to the lack of observations while this is made feasible by fusing data from the other two regions.

## 4. Discussion

Our data fusion approach allows us to combine information from multiple sources that can together describe dynamic and multifactorial phenomena more comprehensively than a single source alone. Infectious disease dynamics are ideally suited for such integrative modeling of an outbreak in which a county is usually affected by its neighboring counties, especially in populated areas, due to population mobility [19]. As the re-emergence of pertussis in the U.S. and Europe in recent years has shown, it is important to have the modeling capacity to predict the incidence of the disease even if the data are usually of small size, which are in themselves not adequate for the precise estimation of tail probabilities.

The multivariate density ratio model described in this study allowed us to examine the joint behavior of pertussis resurgence in adjacent counties. The model was validated by goodness-of-fit plots. Importantly, the observed support of the reference distribution of cases was enlarged by fusing the reference sample with data from nearby regions and applied to the density ratio model. While time series modeling of disease incidence is common in epidemiology, in the face of small or moderate data sources few methods can enhance their input to yield multivariate tail probabilities and confidence intervals, which are not possible to estimate otherwise.

In future work, we plan to further enrich our model with regional covariates to provide key insights for disease surveillance and public health researchers. For instance, the risk factors of pertussis cases that are studied in the U.S. include household size, vaccination coverage and demographics (see Table A1). Such factors are observed with regional variation that is often spatially clustered across neighboring counties [20]. Indeed, data fusion is well suited to the systematic modeling of regions with socioeconomic, political or cultural overlap (e.g., school districts) that are characterized by nonmedical vaccine exemptions, migration and vaccine refusal [21,22,23]. In times of increasingly common vaccine hesitancy, such applications could be very effective for public health.

While the world is currently seeing outbreaks of the COVID-19 pandemic, pertussis is, in comparison, an ancient disease, which was recognized even in the Middle Ages. While connections between these diseases have recently been considered [24], it is beyond the scope of the present study. However, the multivariate approach that we used for fusion of pertussis inter-county data could also be applied to other regionally transmissible diseases, including COVID-19. We leave this to future studies.

## Figures and Tables

**Table 1:** Summary statistics of Jefferson, Cowlitz and Snohomish counties, WA. Q1 and Q3 are referred to 25th and 75th percentile respectively.

	Statistics	Min.	Q1	Median	Q3	Max.
County						
Jefferson		0.00	0.00	1.00	6.50	30.00
Cowlitz		0.00	3.00	8.00	23.25	108.00
Snohomish		7.00	36.25	46.50	54.75	549.00

**Table 2:** Selected joint probability estimates non-obtainable from the empirical distribution and the corresponding 95% confidence intervals. Here, *t* represents the annual pertussis cases in Jefferson.

Probability	Estimate	95% Confidence Interval
P(t>30)	0.0200	(−0.0204, 0.0604)
P(t>40)	0.0084	(−0.0124, 0.0292)
P(t>50)	0.0021	(−0.0041, 0.0083)

**Table 3:** Summary statistics of each county used in the multivariate analysis. Q1 and Q3 are referred to 25th and 75th percentile respectively.

	Statistics	Min.	Q1	Median	Q3	Max.
County						
Grays Harbor		0.00	1.00	2.50	4.75	24.00
Jefferson		0.00	0.00	1.00	6.50	30.00
Clallam		0.00	1.00	2.00	4.75	25.00
Clark		3.00	20.25	33.50	85.00	326.00
Cowlitz		0.00	3.00	8.00	23.25	108.00
Lewis		0.00	2.00	5.00	10.75	71.00
King		38.00	115.00	141.00	194.25	785.00
Snohomish		7.00	36.25	46.50	54.75	549.00
Skagit		1.00	5.00	9.00	17.75	559.00

**Table 4:** AIC values for different choices of $h_{1}$ and $h_{2}$. A hyphen “-” indicates that $h_{k}\left(x\right)\equiv 0$ and therefore $g_{0}$ and $g_{k}$ are identical for k=1,2.

		$h_{1}$	-	$x_{1}$	$x_{2}$	$x_{3}$	$\left(x_{1},x_{2}\right)$	$\left(x_{1},x_{3}\right)$	$\left(x_{2},x_{3}\right)$	$\left(x_{1},x_{3},x_{3}\right)$
	AIC									
$h_{2}$										
-			553.03	554.32	552.37	554.39	554.19	556.22	554.22	556.11
$x_{1}$			527.36	487.62	529.32	526.98	483.92	489.53	528.98	485.89
$x_{2}$			525.03	524.09	516.98	525.37	518.98	525.56	518.94	520.94
$x_{3}$			549.19	551.19	549.92	547.88	551.36	549.45	547.50	549.45
$\left(x_{1},x_{2}\right)$			523.36	485.04	515.77	522.57	485.22	487.03	517.24	487.17
$\left(x_{1},x_{3}\right)$			558.58	489.07	530.52	528.38	485.37	486.05	530.36	483.22
$\left(x_{2},x_{3}\right)$			527.03	526.08	518.97	526.34	520.97	526.85	520.93	522.92
$\left(x_{1},x_{2},x_{3}\right)$			524.91	486.51	517.25	524.33	486.71	483.32	519.19	485.22

**Table 5:** Selected joint probability estimates non-obtainable from the empirical distribution and the corresponding 95% confidence intervals. Here, $\left(t_{1},t_{2},t_{3}\right)$ represents the number of annual pertussis cases in (Grays Harbor, Jefferson, Clallam) respectively.

Probability	Estimate	95% Confidence Interval
$P(t_{1}>20,t_{2}\leq 10,t_{3}\leq 10)$	$5.6511\times 10^{-3}$	$(-2.5641\times 10^{-2}$, $3.6943\times 10^{-2})$
$P(t_{1}>10,t_{2}>10,t_{3}\leq 10)$	$8.1231\times 10^{-3}$	$(-2.9312\times 10^{-2}$, $4.5558\times 10^{-2})$
$P(t_{1}>15,t_{2}>15,t_{3}\leq 15)$	$2.6609\times 10^{-3}$	$(-1.8843\times 10^{-2}$, $2.4166\times 10^{-2})$
$P(t_{1}>25,t_{2}>20,t_{3}>10)$	$2.6517\times 10^{-7}$	($-1.8969\times 10^{-4}$, $1.9010\times 10^{-4}$)
$P(t_{1}>15,t_{2}>30,t_{3}>10)$	$3.7789\times 10^{-9}$	($-2.5683\times 10^{-5}$, $2.5691\times 10^{-5}$)

## Data Availability

Data are available on Washington State Department of Health and U.S. Census Bureau Website.

## Appendix A. Risk Factors of Pertussis Incidence

**Table A1:** County statistics and risk factors: Population Estimate, Average Household Size, Percent Hispanic, Pertussis Vaccine Coverage, Percent Population Below 5 years, Population Density, Rural/Urban, Socioeconomic Status (SES) as per 2017 estimates.

County	Population	Household	%Hispanic	%Vaccine	%Below5	Density	Rural/Urban	SES
Grays Harbor	72,490	2.43	9.8	80.7	5.5	14.78	Mostly Rural	Mid
Jefferson	31,210	2.07	3.7	80.8	2.9	6.70	Mostly Rural	Mid
Clallam	75,637	2.25	5.8	87.1	4.7	16.74	Mostly Rural	Mid
Clark	474,381	2.69	8.7	84.7	6.2	290.74	Semi-Urban	High
Cowlitz	106,805	2.52	8.4	94.1	6.2	36.13	Semi-Urban	High
Lewis	78,320	2.52	9.7	91.5	5.9	12.56	Mostly Rural	Mid
King	2,203,836	2.45	9.4	91.4	5.9	400.75	Urban	Mid/High
Snohomish	802,089	2.68	9.7	90.7	6.4	147.82	Semi-Urban	High
Skagit	125,860	2.55	17.8	90.4	6.1	28.03	Semi-Urban	High
